# Preclinical safety study of nacre powder in an intraosseous sheep model

**DOI:** 10.1136/bmjos-2021-100231

**Published:** 2022-09-15

**Authors:** Donata Iandolo, Norbert Laroche, Dung Kim Nguyen, Miriam Normand, Christophe Met, Ganggang Zhang, Laurence Vico, Didier Mainard, Marthe Rousseau

**Affiliations:** 1 U1059 SAINBIOSE, INSERM, Jean Monnet University, University of Lyon, Mines Saint-Etienne, Saint-Priest-en-Jarez, France; 2 MATEIS, Villeurbanne, Auvergne-Rhône-Alpes, France; 3 88, allée de Signes résidence, Sainte-Baume, Plan-d'Aups-Sainte-Baume, France; 4 Department of Orthopedics, The First Affiliated Hospital of Zhengzhou University, Zhengzhou, China; 5 UMR7365 IMoPA, CNRS/Lorraine University, Nancy, France

**Keywords:** Biomedical Research, Models, Animal, Sheep

## Abstract

**Objectives:**

The purpose of this preclinical study was to evaluate the safety, the local tissue effects and bone healing performance (osteoconduction, osseointegration) of nacre powder in a sheep intraosseous implantation model. This represents the first preclinical study to assess nacre safety and efficacy in supporting new bone formation in accordance with the ISO 10993 standard for biomedical devices.

**Methods:**

The local tissue effects and the material performance were evaluated 8 weeks after implantation by qualitative macroscopic observation and qualitative as well as semiquantitative microscopic analyses of the bone sites. Histopathological characterisations were run to assess local tissue effects. In addition, microarchitectural, histomorphometric and histological characterisations were used to evaluate the effects of the implanted material.

**Results:**

Nacre powder was shown to cause a moderate inflammatory response in the site where it was implanted compared with the sites left empty. The biomaterial implanted within the generated defects was almost entirely degraded over the investigated time span and resulted in the formation of new bone with a seamless connection with the surrounding tissue. On the contrary, in the empty defects, the formation of a thick compact band of sclerotic bone was observed by both microarchitectural and histological characterisation.

**Conclusions:**

Nacre powder was confirmed to be a safe biomaterial for bone regeneration applications in vivo, while supporting bone formation.

**Strengths and limitations of this study**

- Optimisation of the allocations of sites of implantation.
- Reduced use of animals in accordance with 3Rs principles.
- Detailed evaluation of the effects of the material at both local and systemic levels.
- Sample size.
- Single time point.

## Introduction

Autologous cancellous bone of the iliac crest is the material of choice for bone replacement given its good clinical outcomes. However, its use has important limitations, such as its limited availability, the need for an additional surgical procedure and complications with wound healing at the donor site. The development of new synthetic biomaterials is an effective strategic alternative, which has seen continuously growing interest during the past years.^1–5^ In parallel, natural products have been increasingly used to develop bone tissue engineering strategies.

Nacre (mother-of-pearl) is a complex matrix that forms the inner layer of the shell of several species of mollusks (eg, pearl oysters, mussels).

In the search for bone graft substitutes, nacre has increasingly elicited interest as a biomaterial because of its numerous properties.^6^ It was shown to stimulate bone regeneration,^7^ and, through in vivo studies, to be both biocompatible and biodegradable.^8 9^ The ability of nacre to support new bone formation (osteoconductivity) in a bone environment has also been shown in several studies.^10–15^ Moreover, Alakpa and colleagues reported that nacre topography is osteoinductive. Indeed just by using the nacre shell to structure a substrate material, they were able to induce mesenchymal stem cell osteoblastic differentiation.^16^

Like bone, nacre is produced following the deposition of a mineral phase onto an organic matrix. Its brick-like structure is composed of crystallised calcium carbonate in the form of aragonite surrounded by an organic matrix responsible for nacre mechanical properties.^17^ The organic phase of nacre is composed of chitin, proteins, peptides, sugars and lipids and it is the fraction that is recognised to be responsible for its regenerative potential.^18–23^ Several studies have been published that investigate the composition and efficacy of either the water soluble or the ethanol soluble matrices obtained from nacre powder.^19 20^

No preclinical safety study on the use of nacre powder as a bone graft has been performed to date, despite its widespread use in both in vivo and in vitro studies.

In the present work, nacre obtained from the shells of *Pinctada maxima* was adopted, and its biocompatibility was investigated in a sheep model in accordance with the ISO 10993 standard for biomedical devices. The defects were created in both the femoral condyle and the lateroproximal major tubercule of the humerus in sheep. Sheep is a recognised experimental animal model for testing the biocompatibility and local effects of biomaterials to be used as bone substitutes in humans.^24^ We show, with both qualitative and quantitative data, that nacre causes mild inflammatory response in the sheep, while supporting the creation of new bone directly in contact with the old bone.

## Materials and methods (dx.doi.org/10.17504/protocols.io.b44dqys6)

The description of experimental procedures, site allocation and data^25^ analysis has been done respecting the principles of the ARRIVE (Animal Research: Reporting of In Vivo Experiments) guidelines.^26^

### Study design and site allocation

**Figure 1:**
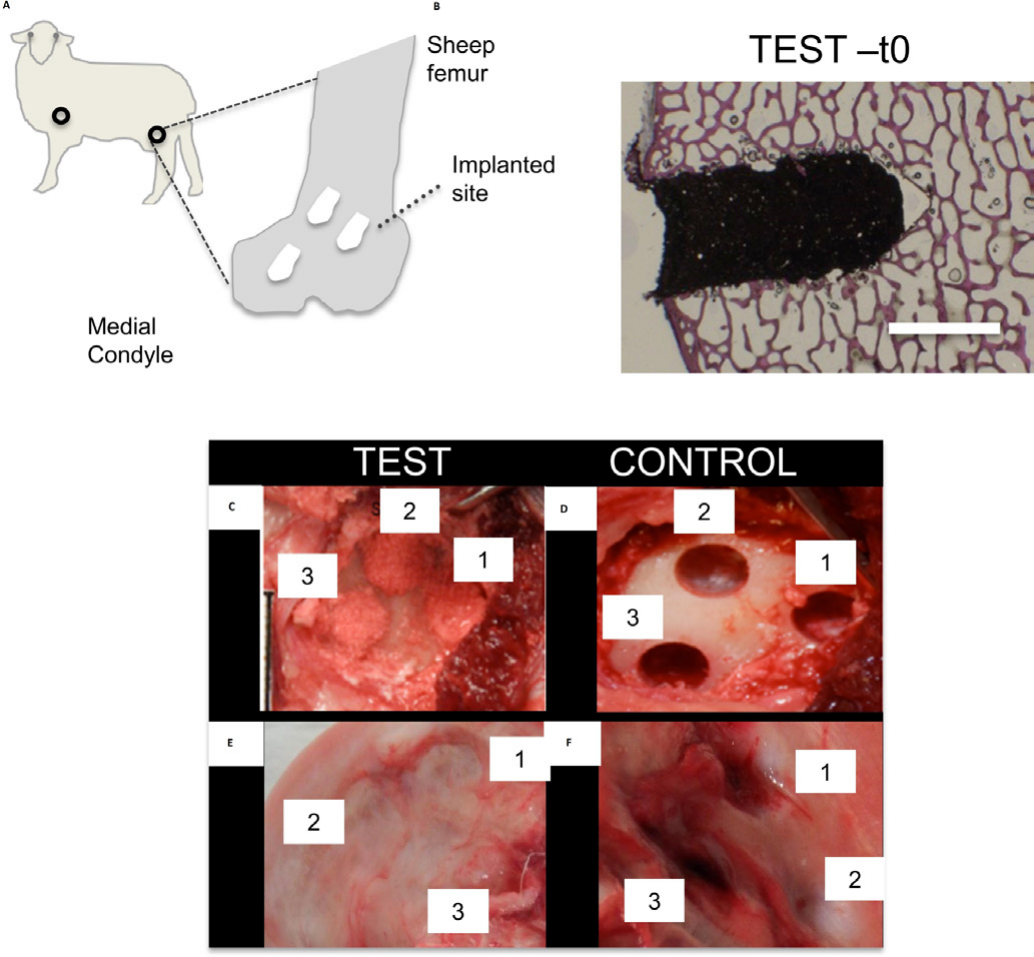
Scheme of the study organisation. (A) Description of the implantation strategy. Empty circles refer to the areas of implantation. Dotted arrow points at the implanted sites within the sheep femur. (B) Histological image of a defect filled with Nacre, stained with the Paragon solution. Scale bar: 5 mm. (C–F) Comparison of the macroscopic appearance of the defect sites at t0 and after 8 weeks from surgery. Defect sites filled with nacre (test) or left empty (control) right after surgery (C,D) and at 8 weeks from surgery (E, F).

Three bone defects (5 mm diameter and 10 mm length) were created bilaterally in the medial part of each femoral condyle and one defect (5 mm diameter and 10 mm length) was generated in the lateral-proximal major tubercle of the humerus of two sheep (figure 1). The sheep were 2.9 and 3.6 years old and they were, therefore, skeletally mature. A total of eight defects were created to test nacre powder and eight used as control samples (randomly attributed as specified in Table 1 and Table 2).

**Table 1:** Approach chosen to guarantee randomisation

Sheep number	Implanted sites/location			
	Left side		Right side	
	Femur	Humerus	Femur	Humerus
Sheep 1	Test (x3)	Test (x1)	Control (x3)	Control (x1)
Sheep 2	Test (x3)	Control (x1)	Control (x3)	Test (x1)

In brackets it is reported the number of implanted sites per area and per condition.

**Table 2:** Overview of analysed samples

Total samples			
Femur		Humerus^#^	
Test	Control	Test	Control
6	6^▲^	2	2

n=6 for the test material and n=5 for the control sites. ▲ One site was not used for the analyses, due to its localisation in the cortical bone. # Data relative to the humerus were not considered for the μ-CT analyses to increase data consistency.

Defects were either filled with the nacre powder (test) (figure 1) or they were left empty (control) according to the site allocation described in table 1.

Sheeps were terminated 8 weeks after surgery.

### Model choice and regulatory aspects

The study was carried out in accordance with the EU Directive 2010/63/EU for animal experiments. The study was also reviewed in accordance with the OECD Good Laboratory Practice regulations, ENV/MC/CHEM (98) 17, with the European Good Laboratory Practice regulations, 2004/10/EC Directive and with the US Food and Drug Administration Good Laboratory Practice regulations, 21 CFR 58. The study was run by the Medical Research Organization NAMSA (Chasse-sur-Rhône, France).

The sheep is an animal model identified for evaluating materials and is recommended in the ISO-10993 standard (part 6, 2007, Biological evaluation of medical devices—part 6: Tests for local effects after implantation) for intraosseous implantations. In addition, a large animal allows for testing relevant size implant material.^24 27^ Moreover, this model is well characterised and it has historically been used in femoral implant studies. In accordance with the ISO-10993 standard, both test material and control were performed in the same animal.

The time period was chosen to evaluate the local tissue effects and the bone healing performance after mid-term implantation (8 weeks), taking into account the kinetics of nacre biodegradation.^12^ Control sites were evaluated to determine the innate healing after 8 weeks in similar defects.

### Surgical procedure

#### Nacre-based paste preparation

Nacre powder (mean particle size: 42.7±5.1 µm), provided by Stansea (Saint-Etienne, France) and produced from the nacreous part of the shells of the pearl oyster *Pinctada maxima*, was sterilised at 121°C for 20 min in an autoclave.^28 29^ Nacre powder was reconstituted as follows: 0.25 mL of autologous blood was sampled and mixed with 1 g of powder just before implantation. The blood was added progressively (drop by drop) and mixed to the powder using a spatula until obtaining a paste to be implanted in the bone defects. The blood/powder ratio was determined during a preliminary feasibility test (data not published).

#### Preoperative procedure

The sheep were fasted approximately 24 hours for food and 12 hours for water before implantation. At the time of implantation, the sheep were weighed and then anaesthetised.

#### Anaesthesia, premedication and preparation of the surgical sites

Premedication was performed by intravenous injection of diazepam (Valium, Roche) and butorphanol (Torbugesic, Zoetis). Anaesthesia was induced by intravenous injection of propofol (Propovet, Abbot Laboratories) and maintained by inhalation of an O_2_—isoflurane mixture (IsoFlo, Axience, 1–5%). Each sheep was infused with Ringer lactate and received intramuscularly a non-steroidal anti-inflammatory drug (flunixine, Meflosyl, Pfizer) and an antibiotic (amoxicillin). The surgical areas were clipped free of wool, scrubbed with povidone iodine (Vetoquinol), wiped with 70% isopropyl alcohol, painted with providone iodine solution (Vetoquinol) and draped. The vital parameters of the sheep were monitored throughout surgery, which was performed by an experienced veterinary surgeon using standard aseptic techniques.

#### Implantation procedure

The sheep was placed on its back. During surgery, a rectal temperature probe and a rumen tube were placed. ECG and oxygen saturation were monitored. The sheep was infused with electrolyte solution (Ringer Lactate, Baxter) to maintain isovolumetric conditions.

#### Surgical approach to the femoral condyle

A cutaneous incision was made on the medial side of each femoral condyle. The vastus medialis muscle was retracted to access the femur. The periosteum was carefully removed from the femoral epiphysis to expose the implant sites.

#### Surgical approach to the humeral major tubercle

A skin incision over the shoulder joint was made from the acromion to the middle of the proximal third of the humerus on each lateral humeral major tubercle. Subcutaneous tissues and deep fascia were dissected and muscles were split longitudinally from the deltoid muscle space, and then the muscle fibres were retracted with blunt dissection. The interspinalis muscle was then retracted caudally by blunt dissection. The periosteum was carefully removed to expose the implant sites. The drill was placed centred in the groove of the humeral major tubercle.

#### Creation of the defects and implantation of the article

Defects with a diameter of 5 mm and a depth of approximately 10 mm were drilled. Drilling was accompanied and followed by extensive rinsing with saline solution to control any temperature increase at the implantation site and to remove bone debris. In the femoral condyle, the sites were spaced by at least 3.0 mm. The defects were cleaned with sterile saline before implantation to avoid any blood clot at the bottom of the defect. The created bone defects were filled with the graft material or left empty (control) (figure 1C,D).

#### Closure of the implanted sites

The incision was closed by suturing both capsule and muscles with absorbable thread (PDS II 1, Ethicon). The subcutaneous layer was closed with absorbable thread (Vicryl 2.0, Ethicon). The skin layer was closed using surgical staples. The wounds were disinfected using an iodine solution (Vetedine solution, Vetoquinol) and then sprayed with oxytetracycline (Oxytetrin spray, Intervet). The operated legs were not restrained in any manner.

### Postoperative procedures

The sheep were left to recover from the anaesthesia in the operating room and returned to their individual cages and kept under close observation. An intramuscular injection of buprenorphine was administered at the end of the surgery day, then daily for 2 days postsurgery. An anti-inflammatory drug (flunixine) was administered daily for 5 days postsurgery and an antibiotic (amoxicillin, Duphamox LA, Zoetis, long action) was given every other day for 8 days following surgery. The surgical staples were removed after complete healing (2 weeks following surgery). The wounds were disinfected with oxytetracycline (Oxytetrin spray, Intervet) every other day until 2 days after the removal of the surgical staples. After this period of recovery, the sheep returned to a farm setting.

#### Termination

At 8 weeks, the sheep were weighed and then euthanised by a lethal intravenous injection of a pentobarbital solution. One additional defect (4 mm diameter) was created in the tibial plateau of one sheep as described before and filled with the nacre paste, to be used as reference sample (t0) for all characterisations. The implanted sites of each sheep were harvested and fixed in 10% neutral buffered formalin.

### Histopathological analysis

After complete fixation, all samples (n=6 for test sites, n=5 for control sites and n=1 for t0) were dehydrated in alcohol solutions at increasing concentration, cleared in xylene and embedded in polymethylmetacrylate (PMMA). One central longitudinal section was obtained by a microcutting and microgrinding system (EXAKT System—thickness of each section ranging between 30 µm and 40 µm) and it was stained with Paragon. Slices of PMMA-embedded samples were stained with both Safranin/Fast Green to detect cartilage and the modified Goldner’s trichrome method to account for the mineralised tissue and osteoid. Images were acquired using a DMRB Microscope (Leica).

### Qualitative and semiquantitative analyses

Qualitative and semiquantitative histopathologic evaluation of the local tissue effects and the performance was conducted for each selected site by anatomopathologists from NAMSA. The analysis was conducted according to the ISO 10 993–6 standard. The following parameters were graded from 0 to 4: cellular inflammatory parameters (polymorphonuclear cells, lymphocytes, plasma cells, macrophages and giant cells/osteoclastic cells); necrosis fibrosis (ultimate inflammatory stage, characterised in histology by an organised deposit of mature collagen); neovascularisation; fatty infiltrate/bone marrow; fibrin; osteolysis and tissue degeneration. The irritation score of the test and control groups was calculated as described in ISO 10993, part 6, Annex E. It corresponded to the sum of the tissue damage and cellular inflammatory parameter scores (eg, lymphocytes, macrophages, Giant cells/osteoclastic cells) weighted with a factor 2, plus the inflammation scores of the repair phase (eg, fibrosis, neovascularisation and fatty infiltrate and bone marrow).

The Irritant Ranking Score (IRS) reflecting the inflammatory reaction and the local tissue effects were determined by subtracting the irritation score of the control from the score of the test article. A negative difference was recorded as zero. The IRS was graded as non-irritant (0.0 to 2.9), slightly irritant (3.0 to 8.9), moderately irritant (9.0 to 15.0) or severely irritant (>15.0). The following parameters were graded from 0 to 4: cellular inflammatory parameters (polymorphonuclear cells, lymphocytes, plasma cells, macrophages and giant cells/osteoclastic cells); necrosis fibrosis (ultimate inflammatory stage, characterised in histology by an organised deposit of mature collagen); neovascularisation; fatty infiltrate/bone marrow; fibrin; osteolysis and tissue degeneration and any other relevant parameters.

The T0 site served as a reference for structural characterisation of the test article.

### Micro-CT

Scans of the PMMA-embedded femurs and humeri were acquired using a micro-CT (Viva CT40, Scanco Medical, Bassersdorf, Switzerland). The scanning parameters were set at 70 kV, 114 µA, 250 ms and the voxel size at 10.5 µm. Three-dimensional reconstructions were generated using the following parameters: sigma=1; support=2; threshold=225.

### Statistical analysis

Median as well as average and SD were calculated for most of the analysed parameters using GraphPad Prism V.5.03 for Windows (GraphPad Software, La Jolla California www.graphpad.com). Samples were compared with the non-parametric Mann-Whitney U test. Significance level was set at 0.05.

## Results

This was the first preclinical study to achieve a thorough characterisation of nacre safety run in the framework of the ISO guidelines for the biological evaluation of medical devices. The study randomisation was achieved by implanting both controls and tests in the same animal, as recommended by the ISO-10993-Part 6 standard.

Defects were created in both sheep’s limbs to avoid a position effect (figure 1A). The total number and distribution of defects per type and sheep is summarised in tables 1 and 2. The depth of the defects (mean±SD) was 10.0±0.0 mm for the test group and 10.3±1.1 mm for the control group. Very slight bleeding of the defect was observed before implantation but it did not impact the implantation procedure. Both sheep gained weight during the study (table 3), with a mean increase between implantation and termination at 8 weeks of 8% and 10%, respectively.

**Table 3:** Age and body weight change of the sheep at implantation and termination (after 8 weeks from surgery).

Sheep number	Age	Body weight at implantation	Body weight at termination	Change in body weight at termination	
	(year)	(kg)	(kg)	(kg)	(%)
Sheep 1	2.9	73	80	7	10
Sheep 2	3.6	79	85	6	8

Figure 1B represents sample test at t0, highlighting the close connection between the material (prepared as described in the Materials and methods section) and the defect site. Macroscopic representative pictures of the different samples at both implantation and termination are presented in figure 1C–F. Transient swellings of the hind leg joints were observed for both test and control joints. These features are common following such periarticular surgery and were not attributed to the implanted graft material. No swellings were observed at termination, confirming that they were related to the immediate, expected postoperative features.

Macroscopic observations at necropsy highlighted a difference mainly in the extent of the area of depression around the created defect (table 4). As expected, control samples displayed a major incidence of marked tissue depressions at the implantation site.

**Table 4:** Macroscopic observations at 8 weeks after surgery for the sham and the test sites

Site of the observation	Macroscopic observations	Occurrence (%)	
		Test	Sham
Implantation sites	Not visible	2/8	1/8
	Whitish/grey colouration of the site	7/8	4/8
	Thin and translucent tissue covering the site	8/8	7/8
	Slight depression on the site	2/8	1/8
	Moderate depression on the site	4/8	1/8
	Marked depression on the site	1/8	5/8
	Red colouration in the defect	0/8	5/8
Surrounding tissues	Red/purplish areas/points	5/8	2/8

**Figure 2:**
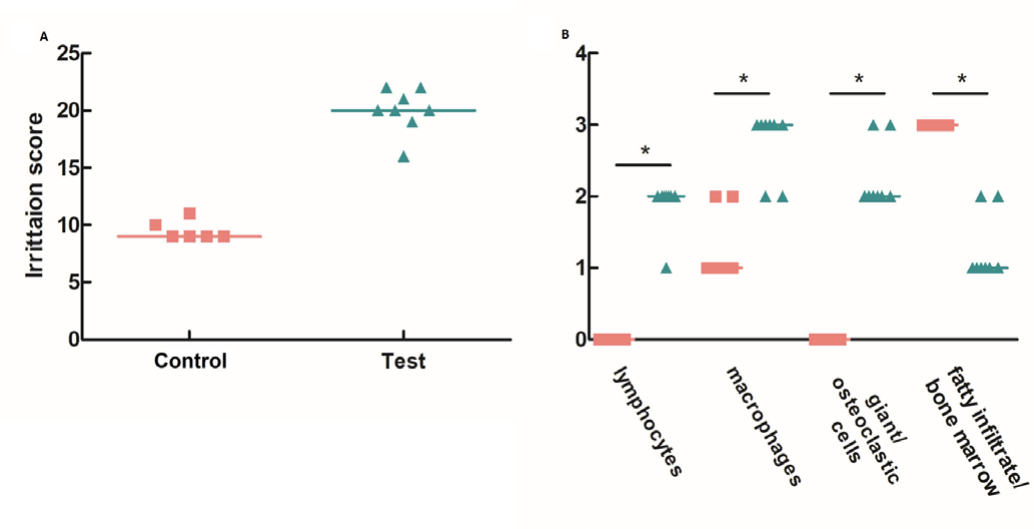
Overall local tissue effects. (A) Dispersion graph of the irritation score calculated for both control (pink squares) and test (green triangles) samples. (B) Dispersion graph of the presence of the cells connected to tissue damage and cellular inflammatory response. The line denoted the median value. *Versus control samples, p<0.05, **Versus control samples, p<0.001.

Histopathological observations were used to define the tissue damage and the cellular inflammatory response (figure 2). Nacre filling was moderately irritant, displaying an irritation score median value of 20.0 compared with 9.5 for the control (figure 2A). IRS value, reflecting the local tissue effects, was calculated and nacre showed to have an IRS index of 10.5. A higher number of lymphocytes, macrophages and giant and osteoclastic cells were found in the sites where nacre had been implanted (figure 2B).

**Figure 3:**
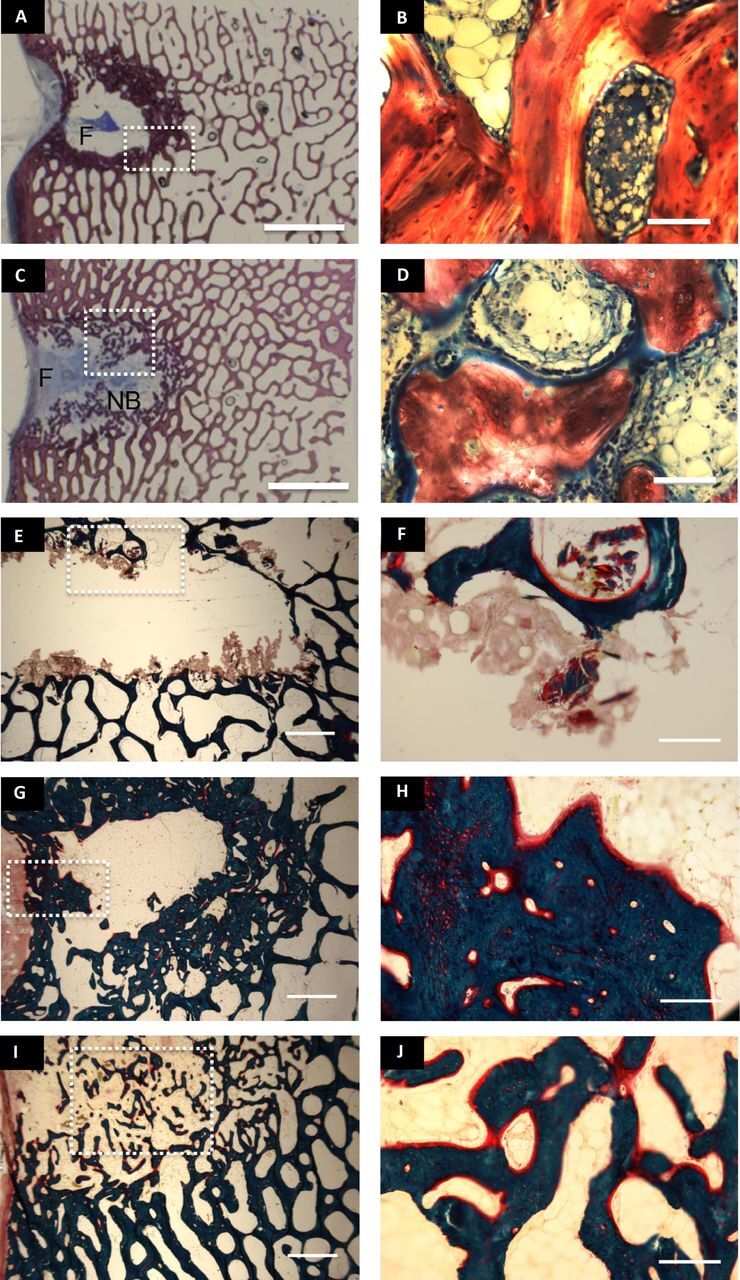
Bone formation and cell response within femoral defects. Paragon staining of PMMA embedded slices of samples from the control (A, B) and nacre-implanted (test) (C, D) defects at 8 weeks. (B) and (D) represent magnifications of details within the dotted areas in figure (A) and (C), respectively. Nb: new bone, F: fibrous tissue. scale bar: (A, C): 5 mm; (B, C): 200 µm. Modified Goldner’s trichrome method of test-t0 (E, F), control at 8 weeks after surgery (G, H), test at 8 weeks after surgery (I, J). Scale bar: (E, G, I): 1 mm; (F, H, J): 200 µm. (F, G and J) correspond to magnifications of the dotted areas in figures (E, G and I), respectively.

**Figure 4:**
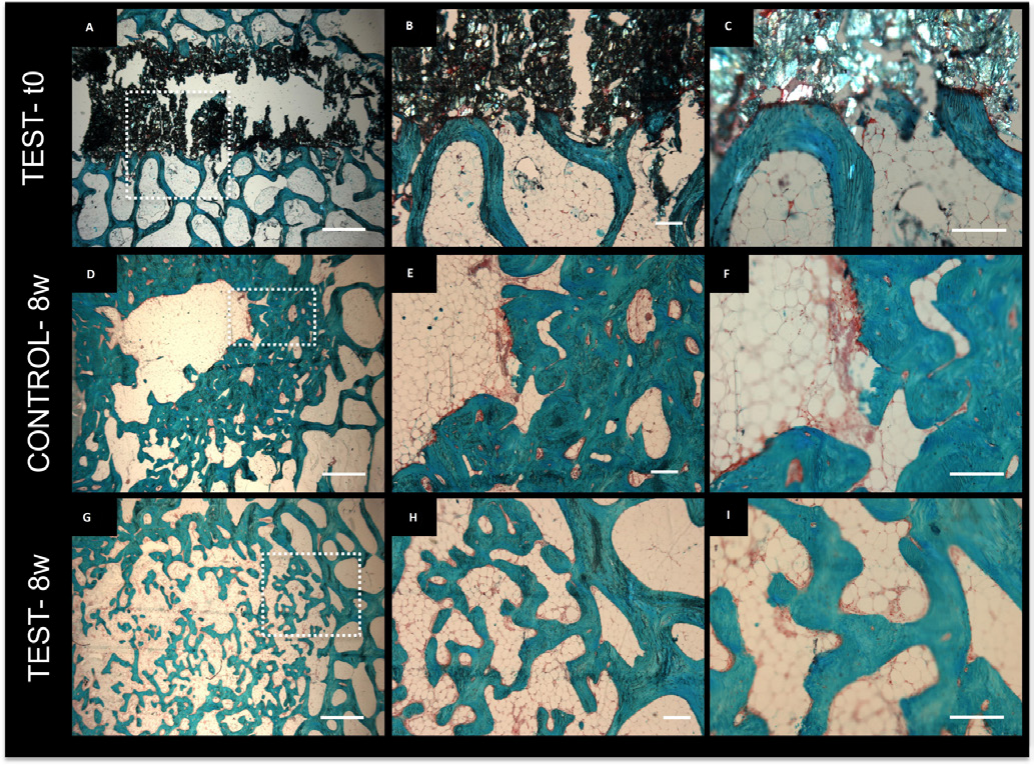
Histological characterisation of the deposited mineralised tissue within the femur. Safranin O/ fast green staining of test-t0 (A–C), control at 8 weeks after surgery (D–F), test at 8 weeks after surgery (G–I) samples. (B and C, E and F, H and I) correspond to magnifications of the dotted areas in (A, D and G), respectively. scale bar: (A, D, G): 1 mm, (B, E, H): 200 µm, (C, F, I): 200 µm. PMMA, polymethylmetacrylate.

Eight weeks after surgery, control defects were mainly filled by fibrotic and adipose tissues (figure 3, figure 4). Deposition of fibrotic tissue accompanied the foreign body reaction to the implanted nacre powder (figure 3C). In the control group, a thick rim of dense, sclerotic bone was noticeable surrounding the defect site (figure 3A). In nacre-filled defects, the newly formed bone was well aligned with the architecture of the older, peri-defect trabeculae and no sclerotic bone formation was apparent (figure 3C). However, the chosen time point was too short to achieve extensive bone formation as it was selected to appreciate long-term biocompatibility of the investigated material. Histological characterisations after trichrome staining (figure 3E–J) revealed a higher extension of new bone formation within the core of nacre-implanted defects compared with those left empty (figure 3). Osteoblastic cells were present within the defect sites for both control and nacre-implanted animals. A more abundant active osteoid was clearly visible for defects filled with nacre, supporting its active role in inducing new bone deposition. No cartilaginous tissue was visible when histological sections were stained with safranin O/fast green (figure 4), suggesting an intramembranous mechanism for the new bone deposition.

**Figure 5:**
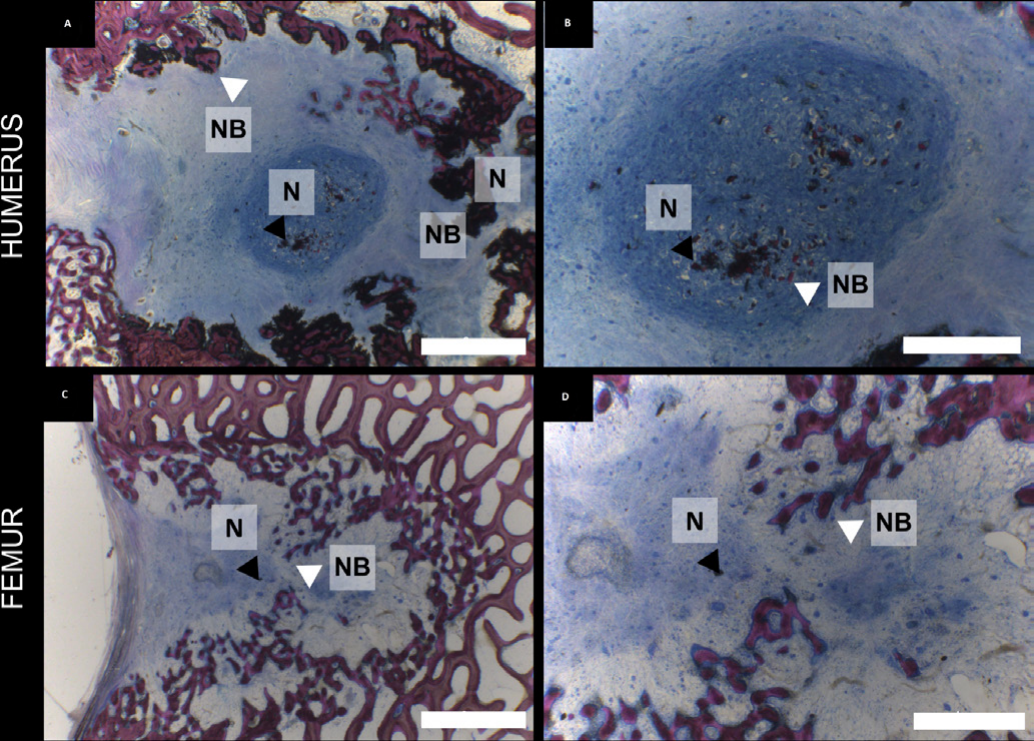
Bone formation within the defect sites. (A–D) Paragon staining of PMMA embedded slices of samples from the humerus (A, B) and femur (C, D) defects at 8 weeks. Scale bar: A and C: 2 mm, B and D: 1 mm. N, nacre, NB, new bone, white arrow head points at newly formed bone around nacre crystals, and black arrow head points at nacre residues.

Small portions of the implanted nacre were still present at termination in humeral defects (figure 5), where new bone was deposited around the nacre particles (figure 5A,B), whereas only traces of the implanted nacre could be found in all femur samples (figure 5C,D).

**Figure 6:**
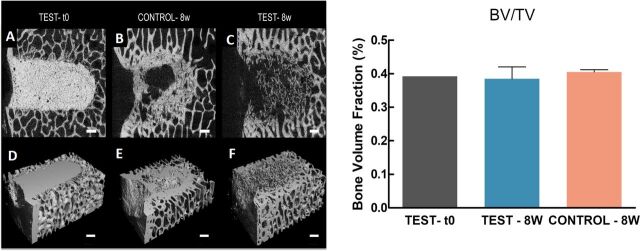
Microarchitectures and none volume fraction (BV/TV) of the defect sites with and without implanted graft material within the femur. (Left panel) (A–C) Micro-CT scans of the femurs of sheep at the defect filled with the nacre paste (test—t0) (A), control 8 weeks after surgery (B) or implanted with the graft material 8 weeks after surgery (C). (D–F) 3D rendering of micro-CT scans of test—t0 (D), control 8 weeks after surgery (control—8w) (E) or implanted with the graft material 8 weeks after surgery (test—8w) (F). Scale bar: 1 mm. (Right panel) Bar chart of the bone volume fraction of the femurs of sheep at the defect filled with the nacre paste (test—t0) (grey), implanted with the graft material 8 weeks after surgery (light blue), control 8 weeks after surgery (dark pink).

Microarchitectural characterisation by micro-CT (figure 6A–C) confirmed a significant difference in the way the new bone had been deposited in the nacre and control group (figure 6B vs C), although no significant differences were evidenced by the analysis of the sample BV/TV (figure 6). In particular, 2D and 3D micro-CT images confirmed the high degree of connection between the old and newly formed bone in sites where nacre had been implanted (figure 6C,F).

## Discussion

Previous works have evaluated the efficacy of nacre in inducing new bone formation both in vitro and in vivo^11 12 30^ as reviewed by Zhang *et al*,^30^ but to the best of our knowledge, this is the first preclinical study run to assess nacre powder biocompatibility in accordance to the ISO standard for the biological evaluation of medical devices.

In our work, a moderate inflammatory response was observed at 8 weeks. Both giant cells and osteoclasts were present within the defect areas, where nacre had been implanted. Giant cells are most frequently associated with a foreign body reaction, and they actively contribute to biomaterial degradation by phagocytosis. Macrophages have been demonstrated to positively contribute to the degradation of nacre, a step necessary for its resorption.^31–33^ It is indeed reported by several groups that erosion by multinucleated giant cells occurs before bone is deposited.^34^ In future studies, this effect will be analysed in comparison to other materials to clearly evaluate the response of our material of choice in the selected host under coherent experimental conditions.

New bone was formed as evidenced by both histology and micro-CT. It is known that cells respond to nacre by depositing a mineralised matrix.^10^ The lack of a complete defect healing is due to the chosen time point. In in vivo studies, longer term time points are preferred to allow for more extensive bone formation,^35 36^ although shorter time points allow to monitor the performances of the material at early stages of the regenerative process. However, the main objective of this study was to prove the material safety rather than its efficacy and an intermediate stage was chosen allowing at the same time the study of the local effects and the biodegradation of the material and early stages of bone formation. The microarchitectural characterisation of the resulting bone at 8 weeks after surgery revealed a significant difference in the degree of connectivity for the two samples as supported by both micro-CT and histological images. The formation of a thick rim of sclerotic bone is a common response to the lack of a support material within the defect.^35 37 38^ In contrast, the implanted nacreous powder supported new bone formation with the creation of seamless bony network between the newly formed osteoid and the old bone (micro-CT and histological characterisations). A nicely anastomosed network of trabeculae was formed within the defect site filled with the nacre powder.^35^ No material was visible at 8 weeks from implantation within the femurs, confirming previous results obtained by Lamghari implanting nacre powder in the vertebrae of sheep.^12^ Moreover, a different degradation kinetic can be suggested for the two different implantation locations, with the femurs allowing for a faster degradation of the nacre powder, compared with the humerus. This result had already been suggested by previous reports as highlighted in the review by Zhang *et al*.^30^

## Conclusions

In this study, sheep was confirmed to be a valid model for in vivo testing of medical devices and in particular of biomaterials. The randomised test confirmed the safety of the studied nacre powder within a time frame of 8 weeks. A moderate inflammation was elicited by the presence of nacre within the created defects. Nacre powder degradation and efficacy in supporting new bone formation were confirmed by the deposition of a network of trabecular bone in continuity with the old bone of the defect site. Our results confirm that nacre is a safe, effective and practical implant material for bone regeneration applications in vivo.

## Data Availability

Data are available in a public, open access repository. The data that support the findings of this study are available at thefollowing link: https://zenodo.org/record/6460395%23.YpoeRJBBxAc.Conditions of reuse: copyright.
